# Metabolite Profiling of External and Internal Petals in Three Different Colors of Tea Flowers (*Camellia sinensis*) Using Widely Targeted Metabolomics

**DOI:** 10.3390/metabo13070784

**Published:** 2023-06-23

**Authors:** Tao Zhang, Xue Ma, Yuanyuan Zhou, Hui Yang, Yuxin Wang, Taolin Chen, Qincao Chen, Yanli Deng

**Affiliations:** 1 College of Tea, Guizhou University, Jiaxiu South Road, Huaxi District, Guiyang 550025, China; taozzz953@126.com (T.Z.); yyhh2y@126.com (H.Y.); wangyuxin6512@126.com (Y.W.); tlchen@gzu.edu.cn (T.C.); 2 College of Agriculture, Jiangxi Agricultural University, No. 1101 Zhimin Avenue, Qingshan Lake District, Nanchang 330045, China; maxue1005@126.com (X.M.); z303225638@126.com (Y.Z.)

**Keywords:** *Camellia sinensis*, tea flower, metabolomics analysis, mass spectrometry

## Abstract

The flower is the reproductive organ of the tea plant, while it is also processed into different kinds of products and thus of great significance to be utilized. In this study, the non-volatile secondary metabolites in the internal and external petals of white, white and pink, and pink tea flowers were studied using a widely targeted metabolomics method with ultra-high liquid chromatography–tandem mass spectrometry (UPLC-MS/MS). A total of 429 metabolites were identified, including 195 flavonoids, 121 phenolic acids, 40 alkaloids, 29 lignans and coumarins, 19 tannins, 17 terpenoids, and 8 other metabolites. The metabolites in the internal and external petals of different colored flowers showed great changes in flavonoids. Most flavonoids and all tannins in the internal petals were higher compared with the external petals. Some phenolic acids were more accumulated in the external petals, while others showed opposite trends. The pink tea flower contained more flavonoids, alkaloids, lignans, coumarins, terpenoids, and tannins compared with white tea flowers. In addition, cyanidin-3-O-glucoside was more accumulated in the external petals of the pink flower, indicating that anthocyanin may be the main reason for the color difference between the pink and white tea flower. The enriched metabolic pathways of different colored flowers were involved in flavonoid biosynthesis, glycine, serine and threonine metabolism, glycerophospholipid metabolism, and phenylpropanoid biosynthesis. The findings of this study broaden the current understanding of non-volatile compound changes in tea plants. It is also helpful to lay a theoretical foundation for integrated applications of tea flowers.

## 1. Introduction

The tea (*Camellia sinensis* [L] O. Kuntze) plant originates from the southwestern region of China and has been cultivated globally, especially in Asian countries such as China, Japan, India, and Thailand, for more than 5000 years [1]. In the tea industry, the tea plant is generally treated as a leaf-use plant, while the tea flower is often regarded as a worthless part of the plant. This results in a great waste of tea flower resources [2], which could provide huge economic benefits if they were used. As the reproductive organ of the tea plant, the tea flower has a showy appearance and can also affect pollination behavior and regulate plant defense response [3]. In addition, the tea flower is directly brewed and drunk after drying in some regions, or is processed into different kinds of products, like tea flower wine and tea flower soap in China [3,4]. Additionally, it is used as food garnish or in drinks in Japan [5]. The tea flower industry also involves cosmetics, functional foods, and other applications.

As a biological organ of tea plants, tea flowers have similar chemical compositions to tea leaves, including abundant flavonoids, polysaccharides, saponins, proteins, and amino acids [6]. Moreover, some recent studies have reported that these bioactive compounds in tea flowers had immense potential in biological activities, such as anticancer, antivirus, and antioxidation [3,7]. Apart from the biological activities above, tea flower extraction can also be used as an intestinal homeostasis regulator and immune stimulator [8]. In addition, tea flower extraction has exhibited antiproliferative activities against human digestive tract carcinoma HSC-2, HSC-4, MKN-45, and Caco-2 cells [9]. The antiproliferative activities of the saponins, such as chakasaponins I, chakasaponins II, and floratheasaponin A, were more potent than those of catechins, flavonoids, and caffeine [10]. The inhibition-induced apoptosis of tea flower extraction was via endogenous rather than exogenous pathways by inhibiting the growth and proliferation of ovarian cancer cell [11]. Moreover, the saponins of the *Camellia japonicum* flower can significantly inhibit the proliferation and dryness of OCSLCs, and inhibit the activation of the Wnt/β-synprotein signaling pathway [12]. These studies imply that tea flowers have broad application values.

The taste, health benefits, and color of tea flowers are generally dominated by the non-volatile components [13]. Compared with tea leaves, many metabolites are more abundant in tea flowers, such as terpenoid compounds, sugars (like glucose, galactose, sorbate, and fructose), organic acids (like shikimic acid, gallic acid, quinic acid, and fumaric acid), and amino acids (like proline, alanine, serine, threonine, valine, n-leucine, and phenylalanine), while catechin gallate, epicatechin, proanthocyanidin B2, quercetin, cunninghamine, and myricetin in tea flowers are lower than that in tea leaves [2,14]. Tea tree flowers contain catechins and theanine, and it was found that theanine content accounts for about 50% of the total free amino acids [15]. EGCG is the most abundant catechu in tea plant flowers. Tea flowers can also be used as a source of hypocaffeine because of their low content of caffeine [16]. In a comparison with other tea species (*C. japonica*, *C. tenuifolia*, 2 savoury *Camellias*, and *C. synaptica*), EGCE and EGC were only detected in tea flower (*Camellia sinensis*) [17]. There are also different flavonoid components between tea flowers of different varieties [18]. Zhou found that a significant difference in flavonoid levels resulted in a difference in the color of golden-flower tea [19]. In addition to non-volatile compounds, there are significant differences in volatile compounds between white and pink tea flowers [20]. The volatile compounds of purple tea flowers are more fragrant than those of white flowers, like methyl eugenol and alpha-ionone [21].

At present, studies on chemical constituents in tea flowers mainly focus on single chemical constituents, such as saponins and tea polyphenols [22,23,24]. The types and contents of chemical components in tea flower have not been presently clarified, which is an important reason to restrict the deep development and utilization of tea flowers. Ultra-high-performance liquid chromatography–tandem mass spectrometry (UPLC-MS/MS) is suitable for the strict requirements of the detection results of scientific research because of its high detection accuracy and great ability to distinguish isomers [25]. As an accurately qualitative and quantitative tool, it has been used to study other species as well, such as *Camellia nitidissima* [26], *Michelia* crassipes [27], and cherry tomato [28], amongst others.

Although a great deal of progress has been made in studying the metabolic difference of different colors of tea flowers, these studies have focused on a few common ingredients.

Thus, UPLC-MS/MS was used to identify and quantify the metabolites in three different-color tea flowers in this study. Multivariate statistical analysis was used to clarify the differences of secondary metabolites in tea flower petals with different colors. The results can provide valuable information and a data reference for the deep development and utilization of tea flowers as an available resource, food, and ornament.

## 2. Materials and Methods

### 2.1. Chemicals and Reagents

MS-grade methanol, acetonitrile, and ethanol were purchased from Merck Corporation (Darmstadt, Germany). Standard compounds were purchased from BioBioPha Co., Ltd. (Kuming, China) or Sigma Corporation (St. Louis, MO, USA).

### 2.2. Plant Materials

**Figure 1:**
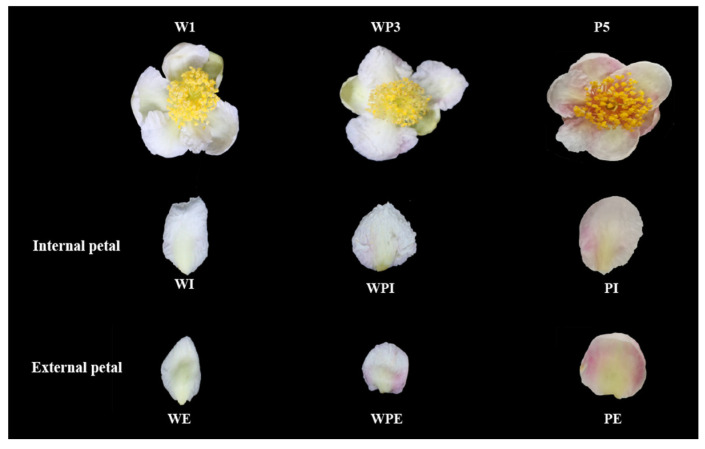
The appearance of tea flower petals.

The tea flowers used in this study were collected in Wangmo County, Guizhou Province (E106°29′49″, N25°35′29″) on 8 December 2021. Wangmo County has an average annual temperature of 16.3 °C and an average annual rainfall of 1339.7 mm. The wild tea trees in this region are community species and have different colors of flowers. White, white and pink, and pink tea flowers were separately collected from different individual tea plants (Figure 1). The external and internal petals in white, white and pink, and pink flowers were labeled as WE, WI, WPE, WPI, PE, and PI. Additionally, when the flowers were more than 75% open, the full blooming stage of tea flower samples was used in the subsequent experiment. After the external and internal petals were separated, the tea flower petals were immediately frozen in liquid nitrogen and stored at −80 °C. Prior to widely targeted metabolomic analysis, tea flowers were freeze-dried with a vacuum freeze-dryer (Scientz-100F, Zhejiang Hengyue Instrument Co., Ltd., Hangzhou, China). The freeze-dried tea flower sample was crushed into homogeneous powder using a mixer mill (MM 400, Retsch, Haan, Germany) with a zirconia bead for 1.5 min at 30 Hz. Dissolved 100 mg powder was extracted with 1.2 mL 70% methanol. Within the first three hours of extraction, the solutions were vortexed for 30 s every 30 min. After that, the solutions were placed at 4 °C overnight. Finally, the solutions were then centrifugated at 12,000 rpm for 10 min. The resulting suspensions were filtered (SCAA-104, 0.22-μm pore size; ANPEL, Shanghai, China) prior to UPLC-MS analysis.

### 2.3. Widely Targeted Metabolic Analysis

Widely targeted metabolomic analysis was assisted by a professional corporation, MetWare (http://www.metware.cn/, accessed on 22 March 2023, Wuhan, China). The extracted non-volatile compounds were separated using an UPLC system (Nexera X2, SHIMADZU, Tokyo, Japan). The UPLC was equipped with an SB-C18 column (1.8 µm, 2.1 mm × 100 mm; Agilent, Palo Alto, CA, USA). Mobile phases A and B were 0.1% formic acid (*v*/*v*) solution and acetonitrile with 0.1% formic acid, respectively. The elution profiles were as follows: 0 min, 95% A and 5% B; 9 min, 5% A and 95% B; 10 min, 5% A and 95% B; 11.1 min, 95% A and 5.0% B; and 14 min, 95% A and 5.0% B. The flow velocity was set as 0.35 mL per minute. The column oven temperature was 40 °C. The injection volume was 4 μL.

The detection of non-volatile compounds was performed on a triple quadrupole linear ion trap/orbitrap mass spectrometer (QQQ-LTQ-Orbitrap-MS, API 4500 QTRAP system, Applied Biosystems owned by Thermo; Carlsbad, CA, USA) equipped with an ESI turbo ion-spray interface, operating in positive and negative ion mode and controlled by Analyst version 1.6.3 (AB Sciex). The ESI source operation parameters were as follows: ion source, turbo spray; source temperature, 550 °C; ion spray (IS) voltage, 5500 V (positive ion mode)/4500 V (negative ion mode); ion source gas I, gas II, curtain gas was 50, 60, and 25.0 psi, respectively; the collision-activated dissociation (CAD) was high. Instrument tuning and mass calibration were performed with 10 and 100 μmol/L polypropylene glycol solutions in the QQQ and LIT modes, respectively. QQQ scans were acquired through MRM experiments with collision gas (nitrogen) set to medium. DP and CE for individual MRM transitions was carried out with further DP and CE optimization. A specific set of MRM transitions were monitored for each period according to the metabolites eluted within this period.

### 2.4. Data Processing

Unsupervised principal component analysis (PCA) was performed by SIMCA version 14.1 (Umetrics AB, Umeå, Sweden), and the data used for PCA were Pareto-scaled (in which the data are centralized and then divided by the square root of standard deviation). The imputed data matrix contained all tea flower samples and respective replicates (*n* = 3) as rows and metabolites as columns. The hierarchical cluster analysis (HCA) results of sample dendrograms were generated by the cofunctions in R (www.r-project.org, accessed on 22 March 2023). The filter criteria for differential metabolites between two groups was as follows: the *p*-value was less than 0.01 and the fold change was greater than 1.5 or less than 0.67. The orthogonal projection to latent structures discriminant analysis (OPLS-DA) was performed by R software (http://www.r-project.org/, accessed on 22 March 2023). Differential metabolites were calculated by combining *p*-values or fold changes of the univariate analysis with VIP (variable importance plot) scores for the OPLS-DA model. Identified metabolites were annotated using the KEGG Compound database (http://www.kegg.jp/kegg/compound/, accessed on 23 March 2023), and annotated metabolites were then mapped to the KEGG Pathway database (http://www.kegg.jp/kegg/pathway.html, accessed on 23 March 2023). Pathways with mapped differential metabolites were then fed into MSEA (metabolite sets enrichment analysis), and their significance was determined with the hypergeometric test. The Heat maps, Venn plot, and K-Means graphs were generated using R online software (http://www.r-project.org/, accessed on 22 March 2023).

## 3. Results

### 3.1. Overall Characterization of Non-Volatile Metabolites

**Figure 2:**
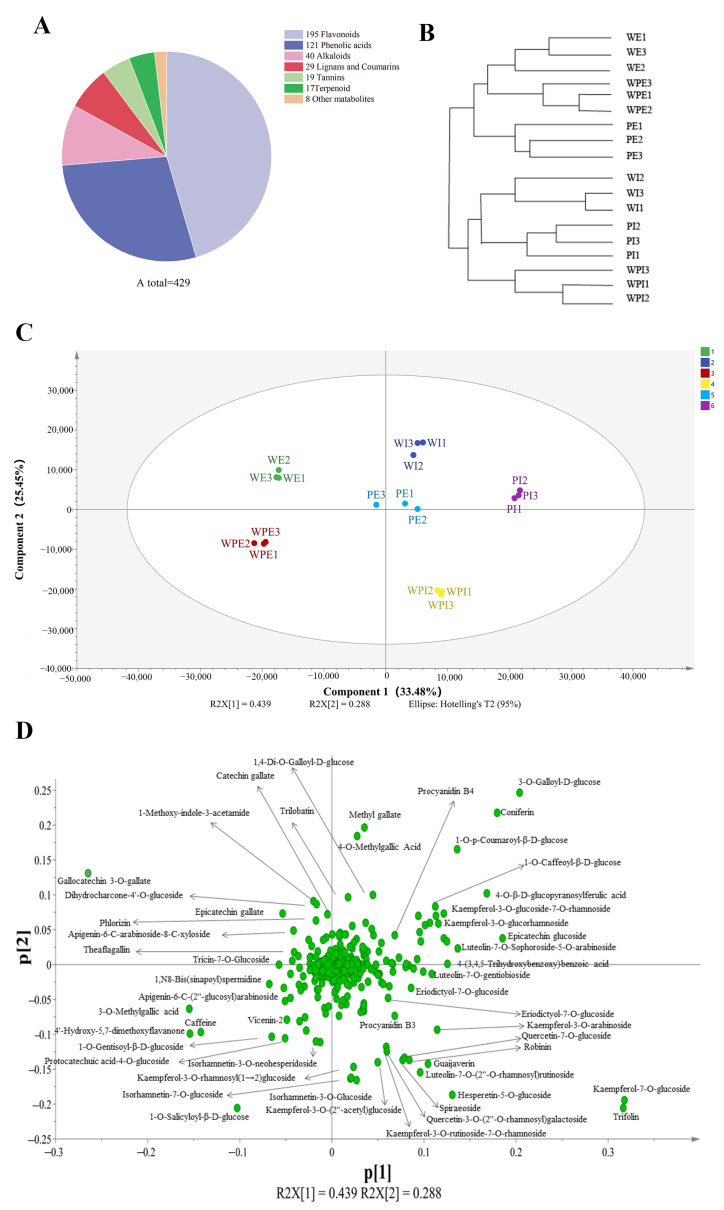
Multivariate statistical analysis of non-volatile secondary metabolites in tea flower samples. (**A**) The chemical composition of metabolites; (**B**) HCA of the internal and external petals; (**C**) score plot for PCA model; (**D**) loading plot of PCA model.

In this study, non-volatile secondary metabolites in the external and internal petals of white, white and pink, and pink tea flowers (labeled as WE, WI, WPE, WPI, PE, and PI) were investigated using an UPLC-QQQ-LTQ-Orbitrap-MS. After comparing the RT and MS information of each peak to that in the MetWare MS database, a total of 429 metabolites were identified, including 195 flavonoids, 121 phenolic acids, 40 alkaloids, 29 lignans and coumarins, 19 tannins, 17 terpenoids, and 8 other metabolites (Table S1 and Figure 2A). HCA was then carried out for six tea flower petals, and the results showed that the external and internal petals were well clustered into two classes (Figure 2B). This implied that the internal and external petals have obvious differences in secondary metabolites.

To obtain an overview of the differences of non-volatile secondary compounds of tea flower samples, PCA was applied using the peak areas of 429 identified compounds after the data were Pareto-scaled. As shown in Figure 2C, the main judgment of the PCA model (R2X = 0.941; and Q2 = 0.863) showed that this model could well illustrate significant differences in metabolites among PE, PI, WE, WI, WPE, and WPI. The first two components of PCA explained 33.48% and 22.45% of the total variance, respectively. It is interesting to note that WE, WPE, and PE were on the left side of the PCA score plot, while WI, WPI, and PI were on the right, which indicated a separation between the external and internal petals and was consistent with the result of HCA (Figure 2B). To further identify the important compounds distinguishing these tea flower samples, the PCA loading plot was generated (Figure 2D). In the PCA loading plot, the dispersion degree of a compound represents its content difference in different groups of samples. As shown in Figure 2D, the metabolites which noticeably contributed to the differences among the six samples were coniferin, catechin gallate, trilobatin, narcissin, procyanidin B4, kaempferol-3-O-glucoside-7-O-rhamnoside, quercetin-7-O-glucoside, 3-O-methylgallic acid, epicatechin gallate, eriodictyol-7-O-glucoside, guaijaverin, theaflagallin, kaempferol-7-O-glucoside, vicenin-2, apigenin-6-C-(2″-glucosyl)arabinoside, 4-O-methylgallic acid, luteolin-7-O-gentiobioside, luteolin-7-O-sophoroside-5-O-arabinoside, luteolin-7-O-(2″-O-rhamnosyl)rutinoside, kaempferol-3-O-rhamnosyl(1→2)glucoside, isorhamnetin-7-O-glucoside, isorhamnetin-3-O-glucoside, and quercetin-3-O-(2″-O-rhamnosyl)galactoside, amongst others.

### 3.2. Comparison of Non-Volatile Secondary Metabolites among the Petals of Three Different Tinctorial Flowers

**Figure 3:**
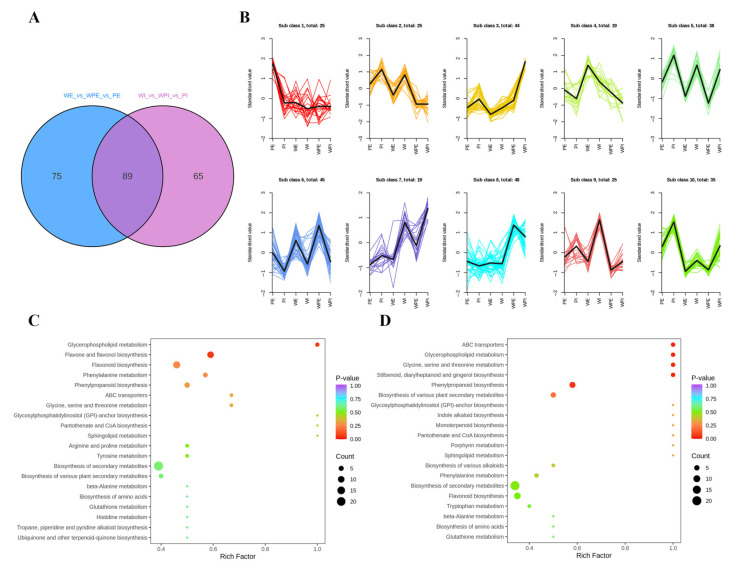
Difference analysis of non-volatile secondary metabolites. (**A**) Venn plot. (**B**) K-means clustering plot. (**C**) KEGG-enriched pathways of WE vs. WPE vs. PE; (**D**) KEGG-enriched pathways of WI vs. WPI vs. PI. The Y-axis on the left represents the KEGG pathway; the X-axis represents “enrich factor”, which is the ratio of DEP number to the total number of annotated proteins in each pathway.

To better understand the metabolite difference among the external or internal petals of different tinctorial tea flowers, the differential metabolites were screened by two criteria: *p* < 0.01 (Student’s *t*-test), and the fold change values being greater than 1.50 or less than 0.67. A total of 225 metabolites showed a significant change, and there were 164 and 154 differential metabolites among the external and internal petals, respectively, and 89 metabolites were mutual in both comparison groups (Figure 3A). The relative content of the differential metabolites was standardized and centralized to study the changing trend of the relative content of metabolites. According to K-means clustering, these differential metabolites among the six tea flower samples were divided into 10 subclasses (Figure 3B). It can be seen that subclass 2, subclass 5, subclass 7, subclass 9, and subclass 10 contained 25, 38, 19, 25, and 35 metabolites, respectively. Additionally, the standardized value of the internal petal was higher than that in the external petal of the three tinctorial flowers in subclass 2, subclass 5, subclass 7, subclass 9, and subclass 10, while subclass 6, containing 45 metabolites, displayed the opposite result. Additionally, WPI showed the highest standardized value in subclass 3 and subclass 7.

For metabolites, KEGG enrichment analysis can better demonstrate the relationship between metabolites and metabolic pathways. In order to understand the internal metabolism relations among the differential metabolites in the external or internal petals of the three different tinctorial flowers, we performed KEGG functional annotation and pathway enrichment analysis. As shown in Figure 3C, differential metabolites among WE, WPE, and PE were annotated in glycine, serine, and threonine metabolism (ko00260); glycerophospholipid metabolism (ko00564); glycosylphosphatidylinositol (GPI)–anchor biosynthesis (ko00563); phenylalanine metabolism (ko00360); phenylpropanoid biosynthesis (ko00940); caffeine metabolism (ko00232); arginine and proline metabolism (ko00330); flavonoid biosynthesis (ko00941); flavone and flavonol biosynthesis (ko00944); and other metabolic pathways, which were involved in the accumulation of important functional metabolites. As shown in Figure 3D, differential metabolites among WI, WPI, and PI were annotated in lysine degradation (ko00310), d-amino acid metabolism (ko00470), indole alkaloid biosynthesis (ko00901), porphyrin metabolism (ko00860), glycolysis/gluconeogenesis (ko00010), isoquinoline alkaloid biosynthesis (ko00950), and monoterpenoid biosynthesis (ko00902). The significantly enriched pathways between the WE vs. WPE vs. PE group and the WI vs. WPI vs. PI group were obviously different, which implied that the metabolic differences among external petals were significantly different from that among internal petals.

#### 3.2.1. Comparison of Non-Volatile Secondary Metabolites among the External Petals

**Figure 4:**
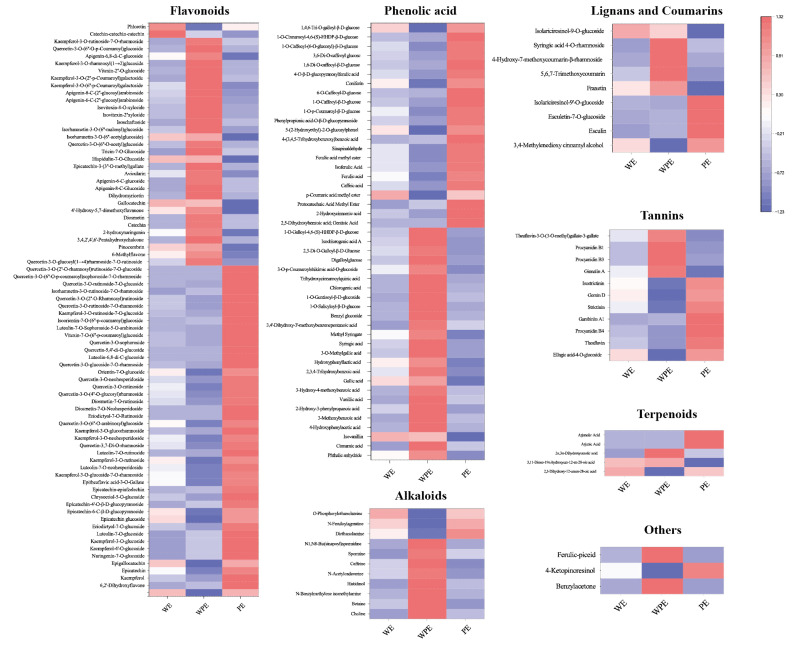
Heatmap of differential metabolites among WE, WPE, and PE.

The levels of differential secondary metabolites among the external petals are shown in Figure 4. As the color changed, the level and variety of metabolites of the three different tinctorial flowers showed great difference.

A total of 78 flavonoids were different among the external petals, among which 45 metabolites were more abundant in PE, including kaempferol-4′-O-glucoside, kaempferol-3-O-glucoside, kaempferol-3-O-glucoside-7-O-rhamnoside, kaempferol-3-O-rutinoside, kaempferol-3-O-neohesperidoside, quercetin-3-O-rutinoside, quercetin-3-O-neohesperidoside, quercetin-3,7-di-O-rhamnoside, vitexin-7-O-(6″-p-coumaroyl)glucoside, luteolin-7-O-sophoroside-5-O-arabinoside, isoorientin-7-O-(6″-p-coumaroyl)glucoside, isorhamnetin-3-O-rutinoside-7-O-rhamnoside, and so on. In total, 25 of 47 phenolic acids were highly expressed in WPE, like phthalic anhydride, cinnamic acid, isovanillin, 4-hydroxyphenylacetic acid, 3-methoxybenzoic acid, 2-hydroxy-3-phenylpropanoic acid, vanillic acid, 3-hydroxy-4-methoxybenzoic acid, gallic acid, 2,3,4-trihydroxybenzoic acid, hydroxyphenyllactic acid, 3-O-methylgallic acid, syringic acid, methyl syringate, and 3,4′-dihydroxy-3′-methoxybenzenepentanoic acid, while others were more accumulated in PE. A total of 11 differential alkaloid metabolites were found, 8 of which were higher in WPE, including choline, betaine, n-benzylmethylene isomethylamine, histidinol, n-acetylcadaverine, caffeine, spermine, N1, and N8-bis(sinapoyl)spermidine. There are 11 different tennis metabolites, 7 of which were more abundant in PE, including ellagic acid-4-O-glucoside, theaflavin, procyanidin B4, gambiriin A1, strictinin, gemin D, and isostrictinin. WPE had more varieties of lignans, coumarins, and terpenoids. In total, the levels and varieties of flavonoids, tannins, lignans, and coumarins in PE were higher.

#### 3.2.2. Comparison of Non-Volatile Secondary Metabolites among the Internal Petals

**Figure 5:**
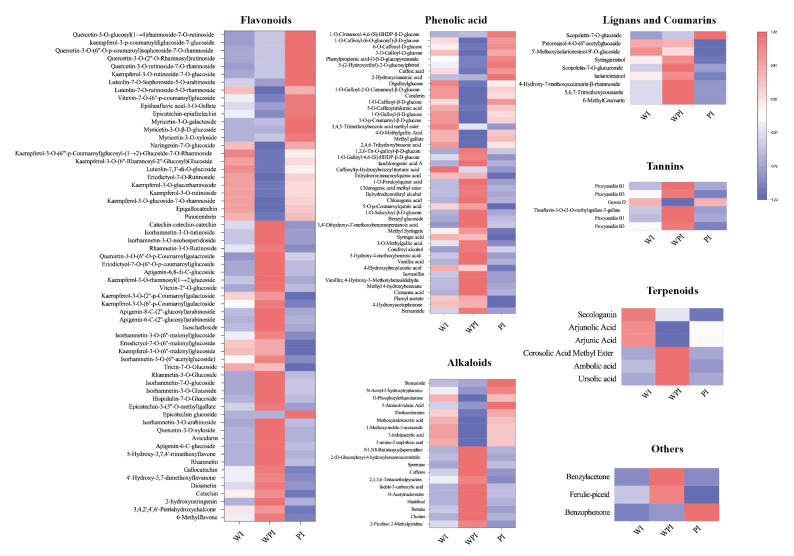
Heatmap of differential metabolites among WI, WPI, and PI.

The levels of differential secondary metabolites among the internal petals of three tinctorial tea flowers are shown in Figure 5. A total of 62 flavonoids were significantly different among the internal petals; 9, 38, and 15 flavonoids showed the highest contents in WI, WPI, and PI, respectively. Pinocembrin, kaempferol-3-O-glucoside-7-O-rhamnoside, epigallocatechin, kaempferol-3-O-rutinoside, kaempferol-3-O-glucorhamnoside, eriodictyol-7-O-rutinoside, and luteolin-7,3′-di-O-glucoside were more accumulated in WI. Most flavonoids, like naringenin-7-O-glucoside, myricetin-3-O-xyloside, myricetin-3-O-β-D-glucoside, myricetin-3-O-galactoside, epicatechin-epiafzelechin, vitexin-7-O-(6″-p-coumaroyl)glucoside, luteolin-7-O-rutinoside-5-O-rhamnoside, and kaempferol-3-O-rutinoside-7-O-glucoside, among others, were more accumulated in PI. The rest of the flavonoids were more accumulated in WPI. In total, 27 of 46 phenolic acids, 11 of 20 alkaloids, 8 of 9 lignans and coumarins, 6 of 7 tannins, 3 of 3 terpenoids, and 2 other metabolites were differentially expressed in WPI, which showed that WPI contained more varieties of metabolites. These mainly consisted of metabolites including benzamide, 4-hydroxyacetophenone, phenyl acetate, cinnamic acid, methyl 4-hydroxybenzoate, vanillin, 2-picoline, choline, betaine, histidinol, scopoletin-7-O-glucuronide, syringaresinol, and so on. It could be concluded that the levels and varieties of WPI were higher than the other internal petals, shown in a different way compared with external petals. Additionally, the tea flowers with pink color contained more flavonoids, like kaempferol, luteolin, quercetin, isorhamnetin, and others. However, WPE and WPI contained more phenolic acids, alkaloids, and tannin varieties.

### 3.3. Comparison of Non-Volatile Secondary Metabolites between the Internal and External Petals of Pink Flowers

**Figure 6:**
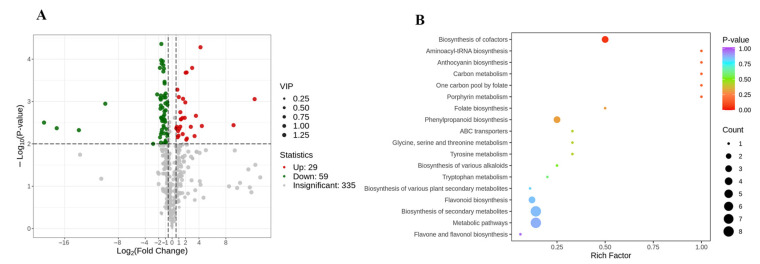
Differential metabolites in the internal and external of pink tea flowers. (**A**) Differentially expressed metabolites between PE and PI. (**B**) KEGG enrichment between WPE and WPI.

As the features of external petals were obviously different from that of internal petals in pink tea flowers, the differential metabolites between the external and internal petals of pink flowers were screened according to the following criteria: FC ≥ 1.5 or ≤0.67; *p* < 0.01, and VIP ≥ 1. In the present study, there were 88 differentially accumulated metabolites between PE and PI (Figure 6A). The KEGG database facilitates the study of metabolites and expression information as a whole network. To more comprehensively understand the relations of differential metabolites between the internal and external petals of pink tea flowers, KEGG functional annotation and pathway enrichment analysis were performed. Most of them were involved in secondary metabolite synthesis, including metabolic pathways (ko01100); biosynthesis of secondary metabolites (ko01110); carbon metabolism (ko01200); biosynthesis of cofactors (ko01240); glycine, serine, and threonine metabolism (ko00260); tryptophan metabolism (ko00380); folate biosynthesis (ko00790); one carbon pool by folate (ko00670); porphyrin metabolism (ko00860); biosynthesis of various plant secondary metabolites (ko00999); aminoacyl-tRNA biosynthesis (ko00970); ABC transporters (ko02010); and so on. Among them, tyrosine metabolism (ko00350), biosynthesis of cofactors (ko01240), and biosynthesis of various alkaloids (ko00996) were involved in accumulation of phenolic acids; and phenylpropanoid biosynthesis (ko00940), flavonoid biosynthesis (ko00941), flavone and flavonol biosynthesis (ko00944), and anthocyanin biosynthesis (ko00942) were relative to the accumulation of flavonoids. Consequently, the different metabolic pathways could probably be the reason for the color changes and metabolite changes of the internal and external petals of pink flowers.

**Figure 7:**
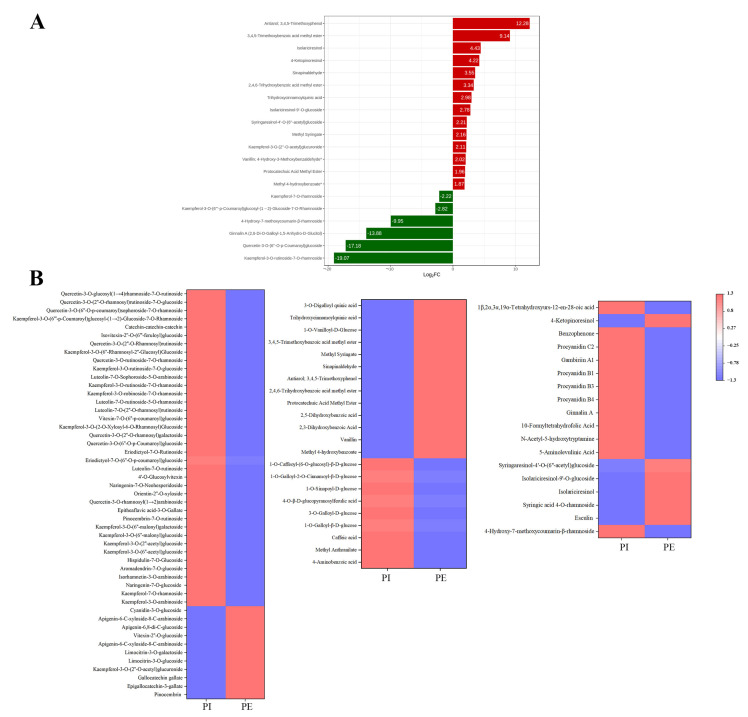
(**A**) Bar chart of top fold change in internal and external petals of pink tea flowers. (**B**) Heat map of internal and external petals of pink tea flowers.

Among the 88 metabolites, 29 metabolites were up-regulated and 59 metabolites were down-regulated (Figure 6A) between the internal and external petals of pink flowers, and the top differential metabolites with high fold change are shown in Figure 7A. The up-regulated metabolites with high fold change were antiarol, 3,4,5-trimethoxybenzoic acid methyl ester, isolariciresinol, 2,4,6-trihydroxybenzoic acid methyl ester, trihydroxycinnamoylquinic acid, isolariciresinol-9′-O-glucoside, syringaresinol-4′-O-(6″-acetyl)glucoside, and methyl syringate; and the down-regulated metabolites with high fold change were 4-hydroxy-7-methoxycoumarin-β-rhamnoside, kaempferol-7-O-rhamnosid, ginnalin A, kaempferol-3-O-rutinoside-7-O-rhamnoside, and kaempferol-3-O-(6‴-p-Coumaroyl) glucosyl-(1→2)-glucoside-7-O-rhamnoside.

As shown in Figure 7B, a total of 48 flavonoids were differentially accumulated and 37 of them were abundantly detected in internal petals, such as prunin, isorhamnetin-3-O-arabinoside, aromadendrin-7-O-glucoside, apigenin-6-C-xyloside-8-C -arabinoside, pinocembrin-7-O-rutinoside, epitheaflavic acid-3-O-Gallate, naringin, luteolin-7-O-rutinoside, eriodictyol-7-O-(6″-O-p-coumaroyl)glucoside, vitexin-7-O-(6″-p-coumaroyl)glucoside, catechin–catechin–catechin, and the derivatives of kaempferol and quercetin. Meanwhile, dihydrochrysin, epigallocatechin-3-gallate, gallocatechin gallate, kaempferol-3-O-(2″-O-acetyl)glucuronide, limocitrin-3-O-glucoside, limocitrin-3-O-galactoside, apigenin-6-C-xyloside-8-C-arabinoside, vitexin-2″-O-glucoside, apigenin-6,8-di-C-glucoside, and cyanidin-3-O-glucoside were more accumulated in PE.

Overall, there were 22 phenolic acids detected and 13 of them were highly accumulated in PI, including methyl 4-hydroxybenzoate, vanillin, 2,3-dihydroxybenzoic acid, gentisic acid, protocatechuic acid, methyl ester, 2,4,6-trihydroxybenzoic acid methyl ester, antiarol, sinapinaldehyde, methyl syringate, 3,4,5-trimethoxybenzoic acid methyl ester, 1-O-vanilloyl-D-glucose, trihydroxycinnamoylquinic acid, and 3-O-digalloyl quinic acid, while other phenolic acids were more accumulated in PI, including 4-Aminobenzoic acid, methyl anthranilate, caffeic acid, 1-O-galloyl-β-D-glucose, 3-O-galloyl-D-glucose, 1-O-caffeoyl-(6-O-glucosyl)-β-D-glucose, and so on.

Most lignans and coumarins were more abundant in PE. Alkaloids, terpenoids, and tannins, mainly consisting of metabolites like ginnalin A, procyanidin B4, procyanidin B3, procyanidin B1, gambiriin A1, procyanidin C2, 5-aminolevulinic acid, N-acetyl-5-hydroxytryptamine, and 10-formyltetrahydrofolic acid, were both abundantly detected in PI.

## 4. Discussion

Tea flower is bisexual and the complete flower contains the stalk, calyx, corolla, stamen, pistil, and 5–6 petals [3]. With the excavation of germplasm resources, some tea tree flowers with 11 petals have been found, and the corolla size varies with the variety [17]. Color is an important phenotypic trait reflecting flower shape and is also a standard for labeling tea germplasm [29]. Tea flowers generally present a white color, while the flowers of a few tea plants may present pink, light yellow, and green colors [30]. The color of tea flowers depends on the variety, genes, and growth environment [31]. At present, most studies focus on the biochemical components, processing [32], and physiological characteristics of white tea flowers [33]. There are few studies on the phenotypic data and metabolite characteristics of pink tea flowers.

Pink tea flower resources were taken from the Wangmo area, in the Guizhou province of China. According to color difference, the samples tended to form six groups (Figure 1), and the clustering results could well distinguish the internal and external petals of the three tea flowers (Figure 2B,C). Although these tea flowers grow in the same environment, the differences in external traits also cause the differences in internal components. In our study, there were 429 secondary metabolites, including 195 flavonoids, 121 phenolic acids, 40 alkaloids, lignin, and 29 coumarin and terpenoids (Figure 2A). Flavonoids in tea flowers were mainly composed of aglycones, including kaempferol, quercetin, myricetin, isorhamnoside, and catechin, amongst others, and a combination of glucuronylation, glycosylation, and glycosylation [34]. Differential phenolic acids included chlorogenic acid, gallic acid, caffeic acid, ferulic acid, coniferin, vanillic acid, and syringic acid. Flavonoids in plants have many effects, indicating that the high content of secondary metabolites of flavonoids in petals are valuable [35]. The sensory flavor, depending on the metabolites, is one of the key factors to evaluate if it is suitable as a food [36]. Flavonoids, alkaloids, and other flavoring components are influential for flavor in tea drinks, and different compositions of them could bring different flavor characteristics [37]. It was reported that the content of flavonoids in tea flowers was higher than that in other flowers, leading to better biological activity with dosage effect [38]. Tea flowers with higher polyphenol content have better antioxidant activity [39]. The accumulation of its composition and content in different tea flowers has specific differences. We found that pink tea flowers contained more flavonoids, alkaloids, lignans, coumarins, terpenoids, and tannins compared with white tea flowers (Figure 4 and Figure 5), while PE and WPI contained more flavonoids, including kaempferol, quercetin, myricetin, isorhamnoside, and catechin. Those metabolites showed a different trend in internal and external petals, especially in petals with color. PE and WPI contained more flavonoids. Flavonoid compounds also have potential medicinal value, and investigations have shown that ingestion of flavonoids can reduce the incidence of various non-communicable diseases, and some flavonoids also show strong physiological activity in vivo and in vitro [40,41]. Thus, tea flowers with color might be a useful resource for processing as drinks for health promotion or used in substance extraction.

White is the main color of tea flowers. Through the collection of germplasm resources, other colored tea flowers have been gradually found [3]. Via the phenotype, we can preliminarily determine that biochemical components of pink tea flowers are different from other tea varieties. In other plant species, there is a strong correlation between various chemical components and color. Flavonoids mainly control the formation of light yellow or nearly white color and are the accessory pigment, while anthocyanins play a major role in the formation of red, pink, blue, and purple colors [42]. Flavonoids are also the main chromogenic substances of flowers, fruits, and seeds, and they can also affect the elongation of petals and promote the germination of pollen [43]. Flavonoid compounds can act as colorants and make plant color more stable through conjunction [44]. For example, there were also small amounts of delphinin and cyanidin in rhododendrons, and the content of cyanin-3-O-rutin in the petals of pink rhododendrons was six times than that of purple rhododendrons [45]. The anthocyanin content in the petals of 10 species of *Rhododendron alpinus* was correlated with their color in the Snow Mountains of southeast Tibet [46]. The level of safflower A was decreased with the reddened and darkened florets [47]. In another study, orange and white safflower florets contained high levels of saffron A and kaempferol 3-o-β-D-glucoside, respectively [48]. In our study, most flavonoids accumulated in WPI and PE (Figure 4 and Figure 5). The color of tea flowers seems to be an important indicator of the accumulation of metabolites. Anthocyanins were detected in all petals (Figure 7B), and the anthocyanin content in the external petals was significantly higher than that in the internal petals (Figure 7B). It implied that anthocyanins may be the main reason for their pink formation. Previous studies have also pointed out that epidermal cytochrome deposition in different parts of petals is different, resulting in phenotype changes [49]. At present, a backcross between the hybrid varieties of *Camellia japonica* and *Camellia nitidissima* is being cultivated to increase the accumulation of flavonoids and achieve the transfer of the yellow gene and the cultivation of yellow camellia varieties [50]. Therefore, the pink tea flower is likely a special material to achieve the ornamental value of *Camellia sinensis*.

Many secondary metabolites, such as phenolic acids, flavonoids, lignin, and other compounds, are produced by the phenylpropanoid pathway and/or its branch pathways in plants. Phenylalanine and tryptophan are catalyzed to produce cinnamic acid and p-coumaryl coenzyme A [51]. The phenylalanine metabolic pathway can be divided into two branches: the phenylalanine metabolic pathway and flavonoid metabolic pathway; and flavonoid biosynthesis is an important downstream branch of phenyl C metabolism [52]. Naringin plays a central role in the metabolic pathway that forms other isoflavones, flavones, and flavonols during its metabolism [41]. Flavonoid scaffolds also undergo a variety of tailoring reactions, such as glycosylation, methylation, and acylation, to form metabolites with different physicochemical and biological properties, which are catalyzed by flavonoid methyltransferase and flavonoyltransferase, respectively [46]. The metabolic components identified in this study involved multiple metabolic pathways, and the main biosynthetic pathways included tyrosine metabolism, biosynthesis of cofactors, biosynthesis of various alkaloids, phenylpropanoid biosynthesis, and flavonoid biosynthesis.

## 5. Conclusions

In this study, the non-volatile secondary metabolites in the internal and external petals of three different colors of tea flowers were studied using a widely targeted metabolomics method. A total of 429 metabolites were identified in three different colors of petals, of which 195 flavonoids, 121 phenolic acids, 40 alkaloids, 29 lignans and coumarins, 19 tannins, 17 terpenoids, and 8 other metabolites were detected. The metabolites of different petals in the internal and external petals showed great changes in flavonoids, phenolic acids, and tannins. The study also found that cyanidin-3-O-glucoside were more accumulated in PE, indicating that anthocyanin may be the main reason for the color difference between the pink and white petal. At present, the utilization rate is very low and studies on the regulation mechanism of non-volatile compounds of tea flowers may provide theoretical guidance for the metabolites basis of tea flower color and provide new ideas of tea flower processing or further processing substance extraction for tea workers.

## Data Availability

Data are contained within the article and Supplementary Materials.

## Supplementary Materials

The following supporting information can be downloaded at: https://www.mdpi.com/article/10.3390/metabo13070784/s1, Table S1: List of 429 identified non-volatile compounds and their contents in the internal and external petals.
